# Protective Effects of Carotenoid-Loaded Nanostructured Lipid Carriers Against Ochratoxin-A-Induced Cytotoxicity

**DOI:** 10.3390/foods13213351

**Published:** 2024-10-22

**Authors:** Nicola Pinna, Pilar Vila-Donat, Denisia Pașca, Francesca Blasi, Aurélie Schoubben, Lara Manyes

**Affiliations:** 1 Department of Pharmaceutical Sciences, Section of Food Science and Nutrition, University of Perugia, 06123 Perugia, Italy; nicola.pinna@dottorandi.unipg.it (N.P.); francesca.blasi@unipg.it (F.B.); 2 Department of Pharmaceutical Sciences, Section of Pharmaceutical Technology, University of Perugia, 06123 Perugia, Italy; aurelie.schoubben@unipg.it; 3 Biotech Agrifood, Facultat de Farmàcia i Ciències de l’Alimentació, Universitat de València, 46100 Burjassot, Spain; denisia.pasca@umfcluj.ro (D.P.); lara.manyes@uv.es (L.M.); 4 Department of Bromatology, Hygiene, Nutrition, Faculty of Pharmacy, “Iuliu Haţieganu” University of Medicine and Pharmacy, 6 Louis Pasteur, 400349 Cluj-Napoca, Romania

**Keywords:** antioxidant, simulated digestion, mycotoxin, cell line

## Abstract

Ochratoxin A (OTA) is a mycotoxin produced by *Aspergillus ochraceous* and various *Penicillium* species, which are known for contaminating agricultural products and posing significant health risks, which include immunotoxicity. This study aims to evaluate the potential of nanostructured lipid carriers (NLCs) loaded with a carotenoid-enriched extract from pumpkin peel (*Cucurbita maxima* L.) in mitigating the toxic effects of OTA. To address the poor bioavailability and instability of carotenoids, nanoencapsulation techniques were employed to enhance their delivery and efficacy. NLCs were formulated using hydrogenated sunflower oil, pumpkin oil, and soy lecithin using hot high-pressure homogenization. The in vitro study involved co-digesting OTA-contaminated bread with an NLC formulation and assessing the impact of the encapsulated carotenoid on OTA bioaccessibility, bioavailability, and cellular toxicity using Caco-2 and Jurkat T cells. Even though no significant influence was observed on the bioaccessibility and bioavailability of OTA, carotenoid-loaded NLCs exhibited cytoprotective effects by improving cell viability and mitigating OTA-induced toxicity in both Caco-2 and Jurkat T cells. Particularly, the flow cytometry analysis highlighted the ability of carotenoids to mitigate OTA-induced cellular damage by decreasing ROS production and limiting mitochondrial mass changes. The study suggests that the encapsulation of carotenoids in NLCs represents a promising strategy to enhance their protective effects against OTA toxicity, potentially offering a novel approach to food safety and public health protection. The study underscores the potential of nanotechnology in improving the bioavailability and efficacy of natural antioxidants to mitigate mycotoxin-induced damage.

## 1. Introduction

Mycotoxins are a significant concern for food safety and public health. These naturally occurring secondary metabolites are produced by various fungal species within the Ascomycota phylum. Due to their toxicological impact and propensity to contaminate a diverse range of crops, including various cereals, in a manner that is inevitable and difficult to predict, mycotoxins pose significant risks to both human and animal health. Among the various types of mycotoxins, with more than 400 identified to date, the following six are commonly and regularly found in different kinds of food: aflatoxins, trichothecenes, zearalenone, fumonisins, ochratoxins, and patulin [1].

Ochratoxins are produced by numerous *Aspergillus* species (e.g., *A. ochraceus*, *A. carbonarius*, and *A. steynii*) and various *Penicillium* species, and one of the most prominent toxins in this category is ochratoxin A (OTA), a phenylalanyl derivative with the capacity to contaminate numerous agricultural products, such as corn, oats, rice, and beans, as well as animal-derived foods, such as meat and offal. The International Agency for Research on Cancer (IARC) currently categorizes OTA as a Group 2B compound (possibly carcinogenic to humans), and its diverse toxicological effects, observed in both humans and animals, are acute nephrotoxicity, hepatoxicity, immunotoxicity, genotoxicity, neurotoxicity, teratogenicity, and embryotoxicity [2]. It is a mycotoxin that is regulated by the European Union [3].

One of the most common mechanisms through which mycotoxins such as OTA exhibit their toxicological effect at a cellular level is an increase in reactive oxygen species (ROS) generation. This is a phenomenon commonly responsible for increases in oxidative stress, inhibitions of protein synthesis, lipid peroxidation, and severe DNA damage, ultimately resulting in cellular alteration and apoptosis. Counteracting mycotoxin-induced oxidative stress by diminishing or neutralizing ROS production after mycotoxin exposure presents a promising strategy to mitigate their toxicological effects. In this context, natural antioxidants have emerged as potential mitigators against mycotoxin-induced toxicity, since they possess the natural ability to counteract oxidative stress by neutralizing free radicals and ROS. Different kinds of antioxidants have been tested against mycotoxin-induced toxicity [4].

Regarding phytochemicals, it is possible to cite carotenoids, which are natural pigments that can be found in various fruits, vegetables, and plants. They are known for their significant role in human health, acting as powerful antioxidants and offering benefits for vision, skin health, and immune function [5]. The protective effect of some carotenoids against mycotoxins has been evaluated both in vitro and in vivo; for example, astaxanthin has been considered for its protective effects against OTA-induced toxicity [6,7]. Furthermore, recent studies have shown how carotenoid-rich fruits, such as pumpkin, can help mitigate the toxic effects of mycotoxins through different mechanisms [8,9]. Increasing the intake of dietary antioxidants, such as carotenoids, through carotenoid-rich fruits like pumpkin, carrots, or spinach, or using commercially available supplements, can enhance the body’s antioxidant abilities and protection against mycotoxins.

Unfortunately, it is imperative to acknowledge the important limitations related to the assimilation of carotenoids through the daily diet that can severely limit their beneficial properties. Despite being fat-soluble compounds, they are highly susceptible to light- and oxygen-induced instability, and they are renowned for their poor bioaccessibility and bioavailability from natural sources, factors that significantly restrict their in vivo activity. The absorption of carotenoids by enterocytes primarily takes place through passive diffusion, and their emulsification and micellization play pivotal roles in facilitating absorption by intestinal cells [10]. In contrast to the high bioaccessibility characterizing mycotoxins such as OTA, carotenoids face major challenges in exerting their beneficial effects against such mycotoxins in a co-ingestion scenario [11]. Various strategies can be applied to improve the bioaccessibility and bioavailability of carotenoids, including nanoencapsulation, which can increase their apparent solubility and bioaccessibility/bioavailability [12]. Enhancing these factors is crucial to improve their effectiveness in counteracting toxicity associated with mycotoxin exposure.

The aim of this study was to obtain a potential carotenoid supplement composed of GRAS (generally regarded as safe) ingredients capable of improving the physicochemical properties of the contained carotenoids and determine its possible use against OTA toxicity. To do so, a co-ingestion scenario was simulated through an in vitro digestion procedure aimed at assessing how the addition of a lyophilized formulation of nanostructured lipid carriers (NLCs) containing a carotenoid-enriched extract, derived from the peel of pumpkin (*Curcubita maxima* L.), may counteract the toxic effects of OTA after the in vitro co-digestion with contaminated bread. The assessment focused on the influence of the NLC formulation on the cytotoxic effect of OTA, including cellular viability, cell cycle, cell death, ROS production, and change in the mitochondrial mass. To the best of our knowledge, few studies have been published regarding the possibility of introducing pharmaceutical formulations containing carotenoids into the daily diet, making this work a solid foundation for future developments.

## 2. Materials and Methods

### 2.1. Chemicals and Reagents

Pumpkins belonging to the *C. maxima* species (Hokkaido variety) were collected in October 2022 from a local farm in Perugia (Umbria region, central Italy). Hydrogenated sunflower oil (HSO) (VGB 5 ST, free fatty acids 0.07%) was a gift from ADM-SIO (Saint-Laurent-Blangy, France), an ADM company. Monobasic sodium phosphate, soy lecithin, and dibasic sodium phosphate were acquired from VWR (Milan, Italy). Cholic acid sodium salt (99%) was bought from Acros Organics (Geel, Belgium). Ultrapure water was produced by Synergy^®^ UV Water Purification System (Millipore Sigma, St. Louis, MO, USA). D-(+)-trehalose anhydrous was purchased from ThermoFisher (Kandel, Germany). Methyl tert-butyl ether (MTBE) and methanol (MeOH) of HPLC grade were purchased from Carlo Erba Reagents (Milan, Italy). The other solvents (petroleum ether, isopropanol, and hexane) were acquired from VWR (Milan, Italy). Lutein (≥92%) was purchased from Extrasynthese (Genay, France). β-carotene (>97.0%) was obtained from TCI (Tokyo Chemical Industry) chemicals (Toshima, Tokyo, Japan). Zeaxanthin dipalmitate was isolated from a hydroalcoholic extract of goji berries and purified as described in a previous work to be used as a standard [13].

Bread ingredients were the following: barley grain (Biogrà, València, Spain), strength wheat flour (Alteza, Toledo, Spain), fresh yeast (Levanova, Valladolid, Spain), white sugar from Pfeifer & Langen GmbH & Co. (Colonia, Germany), and fine sea salt (Polasal S.A., Alicante, Spain).

MeOH for the HPLC, acetonitrile (ACN), and acetic acid (CH_3_COOH) were provided by Fisher Scientific (Madrid, Spain). OTA standard solutions, 3-(4,5-dimethylthiazol)-2,5-diphenyltetrazolium (MTT), potassium chloride (KCl), potassium thiocyanate (KSCN), sodium sulfate (Na_2_SO_4_), sodium dihydrogen phosphate (NaH_2_PO_4_), sodium chloride (NaCl), sodium hydrogen carbonate (NaHCO_3_), α-amylase (930 U/mg; A3403), urea (CO(NH_2_)_2_), hydrochloric acid (HCl), sodium hydroxide (NaOH), pancreatin (762 U/mg; P1750), pepsin A (674 U/mg; P7000), 2’,7’-dichlorodihydrofluorescein diacetate (H_2_DCFDA), ROS detection kit, phosphate buffer solution (PBS), and bile salts (B8631) were purchased from Sigma-Aldrich (St. Louis MO, USA). Deionized water was generated by a Milli-Q water purification system (Millipore, Bedford, MA, USA). MitoTracker green M7514 kit (Invitrogen), dimethyl sulfoxide (DMSO), MitoSOX for mitochondrial ROS generation kit, and the DMEM were purchased from Thermo Fischer Scientific (Waltham, MA, USA). Annexin V-FITC kit was acquired from Miltenyi Biotec (Bergish Gladbach, Germany). The Cycletest™ Plus DNA Reagent Kit was bought from BD Biosciences (San Diego, CA, USA).

### 2.2. Carotenoid Extraction from Pumpkin’s Peel

Carotenoids were extracted from the peel of pumpkins. The pumpkins were processed according to a methodology previously outlined [13] to obtain dehydrated pumpkin peel powder. Carotenoid extraction was conducted using ultrasound-assisted extraction, following the conditions optimized in a previous paper [14].

### 2.3. Oil Extraction from Pumpkin’s Peel

Once separated from pulp, the peel, filaments, and seeds were subjected to the same dehydration process used for the peels, at 45 °C for 12 h, until a constant weight was reached. Once completely dry, pumpkin seed samples were grounded using a blender. The extraction of the lipid fraction of the pumpkin seeds was achieved using petroleum ether as a solvent in a Soxhlet extractor, in compliance with the AOAC procedure [15]. The extract was dried over Na_2_SO_4_, and then the solvent was evaporated at 40 °C under reduced pressure using a rotary evaporator (Büchi Rotavapor B-480, Essen, Germany). Finally, the recovered oil was weighed and stored at 4 °C until use.

### 2.4. Carotenoid-Extract-Loaded NLCs Production

Hot high-pressure homogenization (5 cycles, 1000 bar) was used to produce NLCs. The lipid phase consisted of HSO (1.1% *w*/*v*), pumpkin oil (0.9% *w*/*v*), carotenoid extract (10% *w*/*v*), and soy lecithin (0.8% *w*/*v*), while a buffer solution (pH = 7) was used as the aqueous phase. This buffer solution was prepared by dissolving 4 mM monobasic sodium phosphate and 4 mM dibasic sodium phosphate in ultrapure water containing sodium cholate (0.3% *w*/*v*). Subsequent to production, the NLCs suspension underwent lyophilization to yield a redispersable powder.

### 2.5. Isolation and Quantification of Carotenoids in Lyophilized NLCs Formulation

The carotenoid extraction from the lyophilized formulation was performed by employing a methodology previously described with slight modifications [16]. Lyophilized NLC powder was resuspended in ultrapure water and combined with 6 mL of a 3:2 (*v*:*v*) hexane/isopropanol mix directly in centrifuge tubes. Each sample was vortexed for 1 min and sonicated for 1 min. The extraction procedure was repeated twice and the collected organic phases, once combined, dried under N_2_ flush and stored at −40 °C until chromatographic analysis. Before injection, dried samples were solubilized in MeOH:MTBE (1:1 *v*:*v*) and filtered through PTFE filters (0.22 µm pore) and then analyzed.

The determination of the carotenoid content in lyophilized NLCs was conducted employing a high-performance liquid chromatograph (HPLC-DAD), utilizing a Thermo Spectra Series pump coupled with a UV 6000 LP DAD (Thermo Scientific, Waltham, MA, USA). Chromatographic separation was executed on a reverse-phase C-30 Develosil column (250 × 4.6 mm i.d., 5 μm, Nomura Co., Kyoto, Japan) using the mobile phase solvents A (MeOH: H_2_O, 97:3 *v*/*v*) and B (MTBE). An optimized gradient program, outlined in detail in a previous publication, was employed [14]. Data acquisition was conducted using Xcalibur software (version 1.1) (Chromatographic Specialties Inc., Brockville, ON, Canada). Quantification of the carotenoids was performed utilizing calibration curves derived from standard solutions of lutein (0.24–5.90 μg/mL), β-carotene (0.51–51 μg/mL), and zeaxanthin dipalmitate (0.95–95.0 μg/mL). The details of this method’s validation have previously been reported [14]. Lutein was selected for quantifying non-esterified carotenoids (expressed as μg lutein equivalents/mg of formulation, μg LE/mg), zeaxanthin dipalmitate for esterified carotenoids (expressed as μg zeaxanthin dipalmitate equivalents/mg of formulation, μg ZDE/mg), and β-carotene for sole β-carotene (µg βC/mg of formulation)

### 2.6. Barley Flour Contamination and OTA Production

Contaminated barley flour was procured through the utilization of *Aspergillus steynii* 20,510 sourced from the Spanish Type Culture Collection [17]. A suspension comprising mycelium and spores of the fungal strain was introduced into 1 L glass jars, each containing 400 g of barley grain.

### 2.7. OTA-Contaminated Bread Production

The control (C) and OTA-contaminated (OTA-cb) breads were produced as explained by Mangiapelo et al. [17]. For OTA-cb, the same amounts of all ingredient used for C were employed, and only a fraction of the 7 g of wheat flour was replaced with contaminated barley flour. All components used in the breads’ production are reported in Table 1.

### 2.8. Mycotoxin Extraction from Contaminated Bread

Extraction of OTA was conducted through the addition of a solvents mixture, MeOH/H_2_O (80:20 *v*/*v*), to 2.5 g of C or OTA-cb. The samples were ground for 3 min using an Ultraturrax (T 18 digital Ultra-Turrax^®^, Staufen, Germany), followed by centrifugation for 5 min at 4500 rpm (Centrifuge 5810R, Eppendorff, Germany). For the HPLC-FLD analysis, the resulting supernatant was filtered using a 0.22 μm syringe filter (Phenomenex, Madrid, Spain) and then collected in glass vials. Each sample underwent triple injections (n = 3). Matrix-matched calibration curves were established by fortifying the C extracts with the OTA standard solution to analyze the OTA-cb. Various concentrations (0.0125 µg/mL–5 µg/mL) were employed to construct the calibration lines. An extraction procedure was also performed on the C, combined with the NCL lyophilized formulation, to verify the absence of mycotoxins in the NLC formulation.

### 2.9. HPLC-FLD Quantitative Analysis of OTA

Quantification of the OTA was conducted using an HPLC system, specifically an Agilent 1100 series (Agilent Technologies, Santa Clara, CA, USA). The instrumentation included an automatic sampler, degasser, quaternary pump, and fluorescence detector (FLD) Agilent 1200 (Agilent Technologies, Santa Clara, CA, USA). Data analysis was performed using the software Agilent OpenLab CDS ChemStation Edition rev. C.01.10 (version 3.2.23). A Kinetex EVO C18 column (150 × 4.6 mm, 5 µm particle size, 100 Å pore size, Phenomenex, Palo Alto, CA, USA) was employed. The isocratic mobile phase for OTA consisted of ACN/H_2_O/CH_3_COOH (55/43/2 *v*/*v*/*v*), operated at a flow rate of 0.8 mL/min, with the excitation and emission wavelengths set at λex = 330 nm and λem = 460 nm, respectively. The column was conditioned for 20 min at 40 °C, and the injection volume was 20 µL for the bread extracts and 40 µL for gastric and intestinal extracts. Matrix-matched calibration curves exhibited high R^2^ values (>0.999), indicating the good linearity of the calibration curves for OTA quantification.

### 2.10. In Vitro Static Digestion

To emulate a realistic context of dietary supplement ingestion, lyophilized NLCs underwent an in vitro digestion employing the following two distinct conditions: in combination with control bread (C + NLCs) and with OTA-contaminated bread (OTA-cb + NLCs). In addition to the conditions above, in vitro digestions were also performed on uncontaminated bread (C) and OTA-contaminated bread (OTA-cb) without the addition of the NLC formulation. To imitate the human digestive process, as proposed by Escrivá et al. [18], an in vitro digestion model was applied. This model comprised the following three discernible phases of digestion: oral, gastric, and intestinal. They were used samples weighting 10 g of ground bread (C or OTA-cb), either alone or in combination with 500 mg of the lyophilized NLCs. The obtained digests were preserved at −20 °C.

### 2.11. Gastric and Intestinal Extract Analysis and Bioaccessibility

To determine the OTA, gastric, and intestinal extracts, with and without the addition of NLCs, the digests were centrifuged at 4500 rpm for 5 min. Filtration was performed through a 0.22 μM syringe filter, dilution 1:2 (*v*/*v*) with MeOH, and subsequent injection into the HPLC-FLD. The OTA’s quantification involved interpolation using matrix-matched calibration curves, which were prepared by spiking gastric or intestinal control bread-digested extracts using OTA standards at different concentrations. The OTA bioaccessibility (%) was calculated as the percentage of mycotoxin initially present in the bread revealed in gastric or intestinal digests. The quantity of OTA (μg) in 10 g of bread was derived from the bread concentration (μg/kg) using conversion factors (×10/1000), while the amount of OTA (μg) in 100 mL of the digest was determined from the digest concentration (μg/L) employing conversion factors (×100/1000).

Bioaccessibility % = digest concentration (µg/L) × 1000/bread concentration (µg/kg)

### 2.12. Cell Cultures

Cultivation of human colorectal adenocarcinoma Caco-2 cells (ATCC HB-8065) was carried out in Dulbecco’s Modified Eagle Medium (DMEM) supplemented with 10% fetal bovine serum (FBS), 1% of 100 U/mL penicillin, and 0.1% streptomycin (100 mg/mL). The incubation conditions were set at a pH of 7.4, 5% CO_2_ at 37 °C, and a 95% air atmosphere with constant humidity. Passages were routinely conducted every 2–3 days using 75 cm^2^ plastic flasks with filter screw caps (TPP, Trasadingen, Switzerland).

Human T lymphoblastic Jurkat cells (ATCC-TIB152) were cultured in Jurkat Roswell Park Memorial Institute (RPMI) culture medium by Biowest (Nuaillé, France) supplemented with 1% penicillin (100 U/mL), 0.1% streptomycin (100 mg/mL), and 10% fetal bovine serum (FBS). Passages were conducted at intervals of 2–3 days in 75 cm^2^ plastic flasks under the aforementioned conditions. For the flow cytometry investigations, 0.5 × 10^6^ Jurkat cells were seeded into 6-well tissue-culture plates and exposed to intestinal digests, diluted to 1/10, in cell media for 7 days, with a final concentration of OTA of 0.364 µM.

### 2.13. Cell Viability Assay

The viability assessment of the differentiated Caco-2 cells, following exposure to various intestinal digest concentrations, was conducted using an MTT assay. Caco-2 cells were seeded into 24-well tissue-culture plates, with 500 μL of a 1 × 10^5^ cells/mL suspension added to each well. The culture medium was renewed every 3 days until day 21, representing the duration required for Caco-2 cells to establish an in vitro gastrointestinal barrier. Three distinct exposure durations (24–48–72 h) were examined using different dilutions of intestinal digests (non-diluted: 1/2; 1/4; 1/8; 1/16; 1/32 *v*/*v*). At the end of each exposure period, the medium containing intestinal digest was removed, and a 2.5 mg/mL MTT solution was introduced to each well. Following a 4 h-incubation at 37 °C, the MTT solution was eliminated, and DMSO was introduced. The absorbance was read at 570 nm with a Biotek Synergy H1 Multimode Plate Reader (Agilent, Winooski, VT, USA).

### 2.14. Bioavailability In Vitro

Caco-2 cells were seeded in the upper compartment of a 6-well Transwell Permeable Support system with a 12 mm diameter (Corning, New York, NY, USA) and a 0.4 mm pore size, utilizing 500 µL of a 4.5 × 10^5^ cells/mL suspension. The cells underwent a 21-day cultivation period, during which complete differentiation was achieved, and the culture medium was renewed every 2–3 days. The monolayer integrity for the two control inserts was evaluated on day 21. The apical (upper compartment) and basal (lower compartment) mediums were removed, and the cells were subjected to three washes with PBS solution. The cells were then transferred to a new plate, and 1300 µL of PBS was introduced into the basal compartment. In the apical compartment, 200 µL of a phenol red solution in PBS at a concentration of 42 μmol/L was added. The plate was incubated at 37 °C for 1 h, following which 100 µL of the basal compartment was collected, and the phenol red content was quantified at 570 nm using a spectrometer. The observation of a phenol red concentration lower than 6% of the initial concentration suggested the integrity of the cell monolayer.

For the Caco-2 exposed to the intestinal digest, they were centrifuged at 4200 rpm for 15 min to collect the resulting supernatant. Subsequently, the apical and basal media were removed, and the cells were washed with a PBS solution. The support was then transferred to a new plate, and 750 µL of PBS was introduced into the basal compartment, while 750 µL of each intestinal digest was added to the apical compartment. Following a 4 h incubation period at 37 °C, the basolateral compartment was collected, freeze-dried overnight, and stored at 4 °C until further analyses.

### 2.15. Cell-Cycle Analysis

The extraction and staining of the Jurkat cell nuclei were accomplished using a DNA reagent kit. Subsequent flow cytometric analysis was used to delineate the distribution of cell-cycle phases. Following the intestinal diges exposure, Jurkat cells were harvested and subjected to centrifugation as per the manufacturer’s instructions. After the addition of propidium iodide stain solution, a final incubation at 4 °C for 10 min in the dark was performed, and the samples were then analyzed using a flow cytometer MACSQuant^®^ Analyzer 16 (Myltenyi Biotec GmbH, Bergisch Gladbach, Germany).

### 2.16. ROS Analysis

At the end of the exposure period, Jurkat cells were harvested, placed in a centrifuge tube, and subjected to centrifugation at 1400 rpm for 5 min. The cellular pellet was reconstituted with a 5 μM H_2_DCFDA solution, followed by a 20 min incubation at 37 °C in the dark. Subsequently, the samples underwent two PBS washes and were then analyzed using a flow cytometer. As a positive control, 1 mM tert-butyl hydroperoxide (TBHP) was employed after a 30 min incubation period.

### 2.17. Mitochondrial Mass and Mitochondrial ROS Analysis

The mitochondrial mass from the Jurkat cells after exposure was assessed employing MitoTracker green dye. Following the various exposures, a population of 1–3 × 10^5^ Jurkat cells was gathered in a centrifuge tube and subjected to centrifugation for 5 min at 1400 rpm. The resulting cell pellet was reconstituted in a staining solution, encompassing a 100 nM MitoTracker probe, and subjected to a 20 min incubation at 37 °C in the dark. After the staining incubation, the samples were centrifugated, reconstituted in PBS, and subjected to analysis using a flow cytometer.

The evaluation of the mitochondrial ROS generation in Jurkat cells was conducted using the MitoSOX reagent (at a final concentration of 1 μM). In this context, 1–3 × 10^5^ Jurkat cells were collected in a 15 mL centrifuge tube and subjected to centrifugation. MitoSOX reagent was introduced to each sample followed by PBS washes, and the samples were subsequently analyzed by flow cytometer. As a positive control, a 50 μM MitoParaquat solution was employed after a 16 h incubation period.

### 2.18. Flow Cytometer Settings

All flow cytometry assessments were conducted utilizing the MACSQuant^®^ Analyzer 16 (Miltenyi Biotech GmbH, Bergisch Gladbach, Germany) equipped with blue (488 nm), violet (405 nm), and red (640 nm) lasers. Fluorescence data for Annexin V, MitoTracker, and H_2_DCFDA were acquired using a 525/50 FITC B1 filter. The MitoSOX fluorescence data were captured using a V4 channel with a 615/20 filter, while the cell-cycle fluorescence data were acquired using a 579/34 PE (B2) filter. A minimum of 20,000 events were considered for each analysis.

### 2.19. Statistical Analyses of the Data

The statistical analysis of the results was performed by applying the Student’s *t*-test for paired samples, with *p* ≤ 0.05 considered statistically significant. Microsoft Excel^®^ for Microsoft 365 MSO (Version 2306 Build 16.0.16529.20164) was utilized as the statistical software. GraphPad Prism version 9.3.1 for Windows (Boston, MA, USA) was used to build the graphs. The software applied for the flow cytometry assays was Macs Quantify version 2.1. Data are expressed as the means ± SD.

## 3. Results

### 3.1. Carotenoid Quantification of NLC Formulation

The results reveal concentrations of LE, β-carotene, and ZDE within the formulation corresponding to 0.017 ± 0.0007 µg LE/mg, 0.0023 ± 0.0001 µg βC/mg, and 0.548 ± 0.0219 µg ZDE/mg. Figure S1 shows the SEM photomicrographs of the NLCs, which are characterized by a spherical shape.

### 3.2. OTA-Contaminated Bread Analysis

The final concentration of OTA in the contaminated bread was 13.01 ± 0.14 mg/kg. No trace of OTA was found in the control bread or NLC formulation. The OTA concentrations in the mycotoxin-contaminated bread, alone and combined with NLCs in the gastric and intestinal digests, are reported in Table 2.

### 3.3. OTA Bioaccessibility in Gastric and Intestinal Digests

The assessment of the OTA bioaccessibility involved applying an in vitro digestion model coupled with HPLC-FLD analysis for quantification in gastric and intestinal digests. Concerning the OTA bioaccessibility, substantial disparities were noted when comparing the gastric and intestinal bioaccessibility of OTA-cb and OTA-cb + NLCs. In both the OTA-cb and OTA-cb + NLCs, the gastric bioaccessibility was markedly lower, at 3.9 ± 0.1% and 3.8 ± 0.2%, respectively, when compared to their respective intestinal bioaccessibility, which stood at 100 ± 1.0% and 90.8 ± 2.5%. No significant differences were observed when compared to the OTA bioaccessibility in the gastric and intestinal digests of the OTA-cb and OTA-cb + NLCs.

### 3.4. Cell Viability

The cytotoxic effects of OTA, alone and combined with the NLC formulation, are described in Table 3. In the context of OTA exposure, the assessment conducted at a 24 h time point revealed no statistically significant differences in the cellular viability of Caco-2 cells exposed to the OTA-cb and OTA-cb + NLC intestinal digests. However, a distinctive pattern was observed at the 48 h and 72 h exposure time points; notably, significant differences in cell viability were observed across all tested dilutions, suggesting improvements in cellular viability when NLCs were present in the intestinal digest, ranging from 21 to 35% for the 48 h period and 9 to 12% for the 72 h period.

### 3.5. OTA Bioavailability Assessment

Unidirectional transport of OTA was evaluated from the apical to basolateral side of the differentiated cell monolayers. The bioavailability (%) was quantified as the percentage of OTA concentration initially loaded into the apical side and subsequently detected on the basolateral side following a 4 h incubation period with OTA-cb and OTA-cb + NLC intestinal digest. The results indicate bioavailabilities of OTA of 29.9 ± 9.6% and 31.1 ± 1.5% for the OTA-cb and OTA-cb + NLC intestinal digests, respectively. This suggests that the presence of the NLC formulation did not impact the OTA bioavailability.

### 3.6. Cell-Cycle Analysis

**Figure 1:**
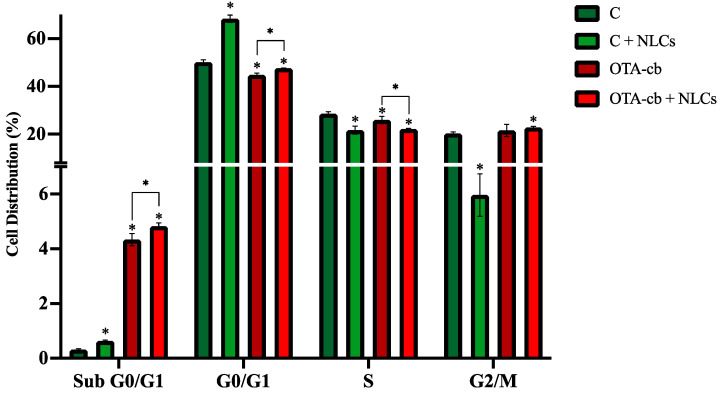
Bread intestinal digest’s (0.330 ± 0.004 µM OTA) effect on Jurkat cell-cycle phases (sub-G0/G1, -G0/G1, -S, and -G2/M). Data are expressed as the means ± SD (n = 4), and significative differences are indicated at *p* < 0.05 (*). Significant differences between the C digest and C + NLCs, OTA-cb, and OTA-cb + NLCs are denoted by asterisks directly above the columns. Significative differences between OTA-cb and OTA-cb + NLCs are indicated by asterisks above the brackets. NLCs, nanostructured lipid carriers; OTA, ochratoxin A; OTA-cb, ochratoxin-A-contaminated bread.

A PI staining kit was utilized to assess the impact of intestinal digests on the cell cycle. The findings, depicted in Figure 1, reveal a pronounced arrest in the sub-G0/G1 phases among cells exposed to C + NLC intestinal digests, as compared to the C, OTA-cb, and OTA-cb + NLC intestinal digest. Additionally, the presence of the NLC formulation in the C + NLC intestinal digest resulted in a significant increase (*p* < 0.01) in cells in the G0/G1 phases and a significant reduction in cells in the S (*p* < 0.05) and G2/M phases (*p* < 0.05), compared to the C intestinal digest. The cells exposed to OTA, whether in the OTA-cb or the OTA-cb + NLC intestinal digest, exhibited a slightly diminished distribution of cells in the subunit G0/G1, as well as an augmented distribution in the G2/M subunit.

### 3.7. Apoptosis/Necrosis Pathway Analysis

**Figure 2:**
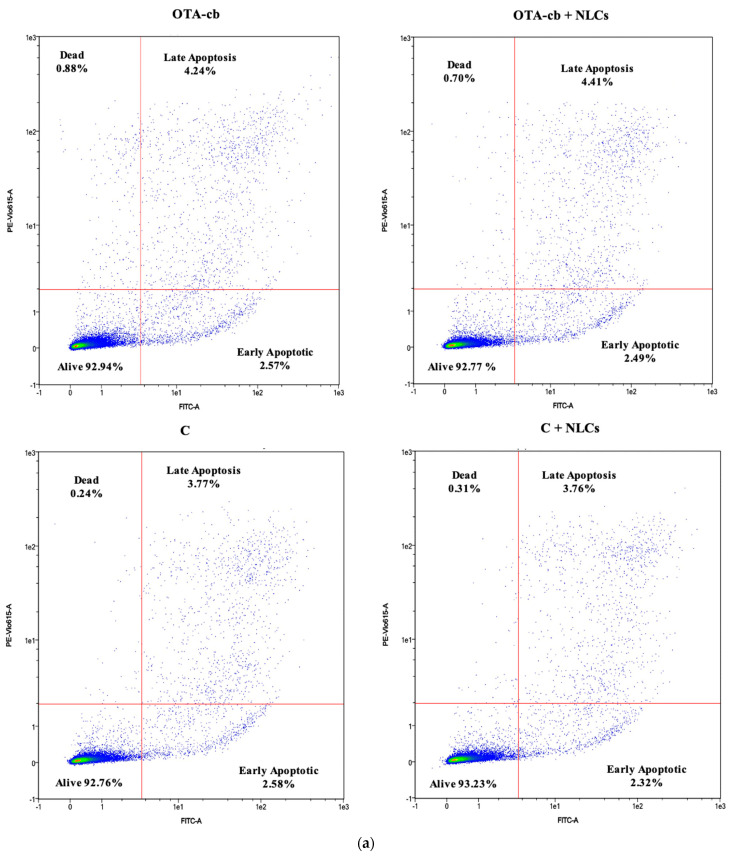

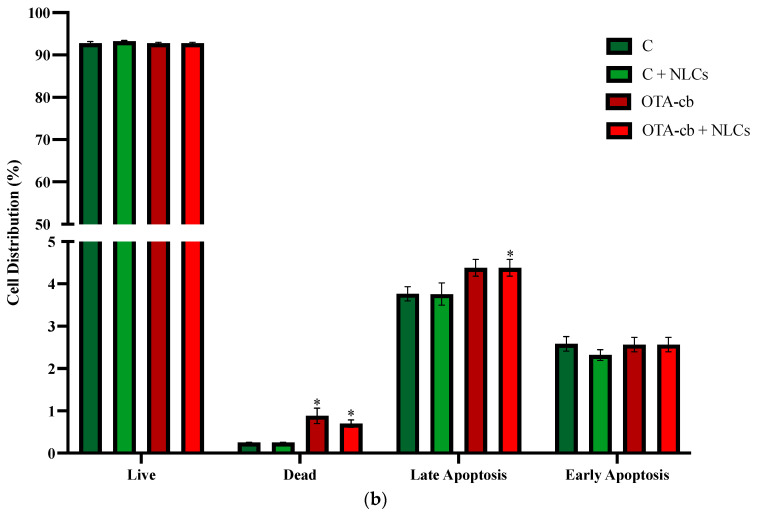
Intestinal digests’ effects (0.330 ± 0.004 μM OTA) on the apoptosis/necrosis pathway: (**a**) plots from the cell apoptosis assay after Jurkat exposure to intestinal digests; (**b**) bar graph reporting the means ± SD (n = 4). Significative differences are indicated at *p* < 0.05 (*). Significant differences between the C digest and C + NLCs, OTA-cb, and OTA-cb + NLCs are denoted by asterisks directly above the columns. C, control; NLCs, nanostructured lipid carriers; OTA-cb, ochratoxin-A-contaminated bread.

The impacts of intestinal digests on the apoptosis/necrosis pathway are illustrated in Figure 2. Following exposure to the C intestinal digest, the results reveal a distribution of 92.7 ± 0.4% viable cells, 0.2 ± 0.0% dead cells, 3.8 ± 0.2% cells in the late apoptosis stage, and 2.6 ± 0.2% cells in the early apoptosis stage. The statistical analysis showed no significant differences in these stages compared to the other intestinal digests, except for a significant increase in the proportion of dead cells (0.9 ± 0.2% and 0.7 ± 0.1%) observed when the cells were exposed to OTA (*p* < 0.001) and OTA + NLC (*p* < 0.01) intestinal digests, as well as an increase in the cell distribution in the late apoptosis stage (4.2 ± 0.3% and 4.4 ± 0.2%).

### 3.8. ROS Analysis

**Figure 3:**
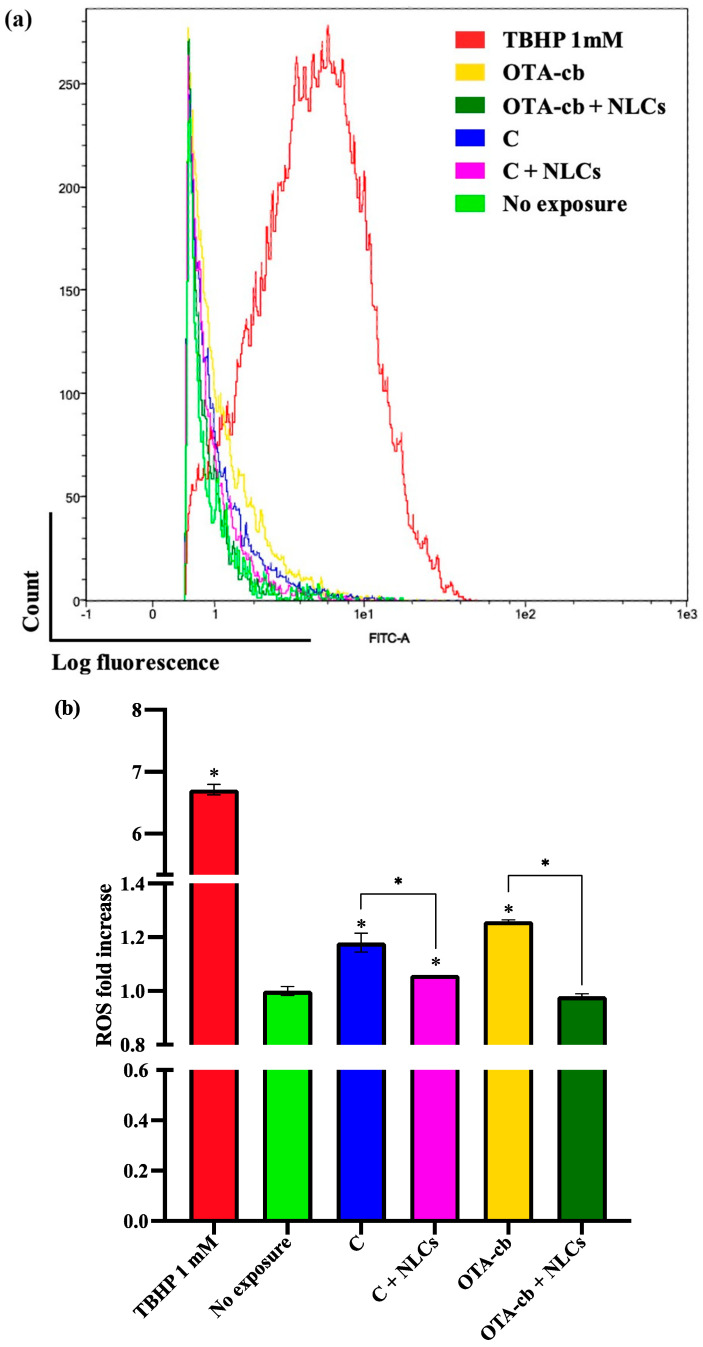
Effects of the intestinal digests (0.330 ± 0.004 μM OTA) on ROS generation: (**a**) representative plots of cell counts versus the LOG fluorescence of 20.000 events, analyzed using a flow cytometer for the detection of ROS; (**b**) bar graph reporting the intensity of the mean fluorescence ± SD (n = 4), expressed as the fold increase in the non-exposed. Significative differences are indicated for *p* < 0.05 (*). Significant differences between the nonexposed cells and cells exposed to the C, C + NLCs, OTA-cb, and OTA-cb + NLCs digests are denoted by asterisks directly above the columns. Significative differences for C vs. C + NLCs and OTA-cb vs. OTA-cb + NLCs are indicated by asterisks above the brackets. NLCs, nanostructured lipid carriers; OTA, ochratoxin A; OTA-cb, ochratoxin-A-contaminated bread.

As depicted in the Figure 3, exposure to both the C and OTA-cb intestinal digests resulted in significant elevations in ROS levels (*p* < 0.01 and *p* < 0.001, respectively) compared to unexposed cells. Conversely, the addition of the NLC formulation, present in both the C + NLC and OTA-cb + NLC intestinal digests, attenuated the ROS increase in the exposed cells, restoring the concentrations to levels comparable to those in unexposed cells.

### 3.9. Mitochondrial ROS Analysis

**Figure 4:**
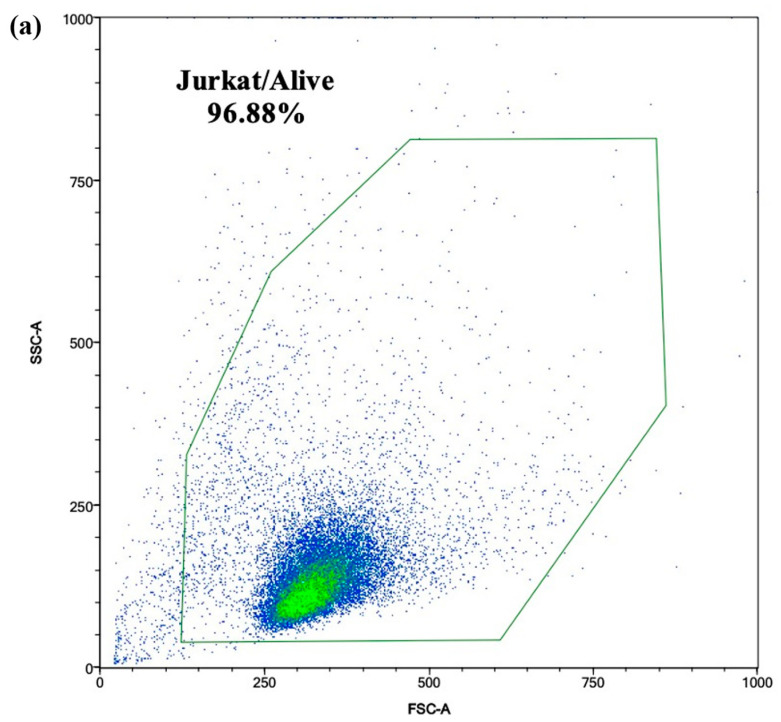

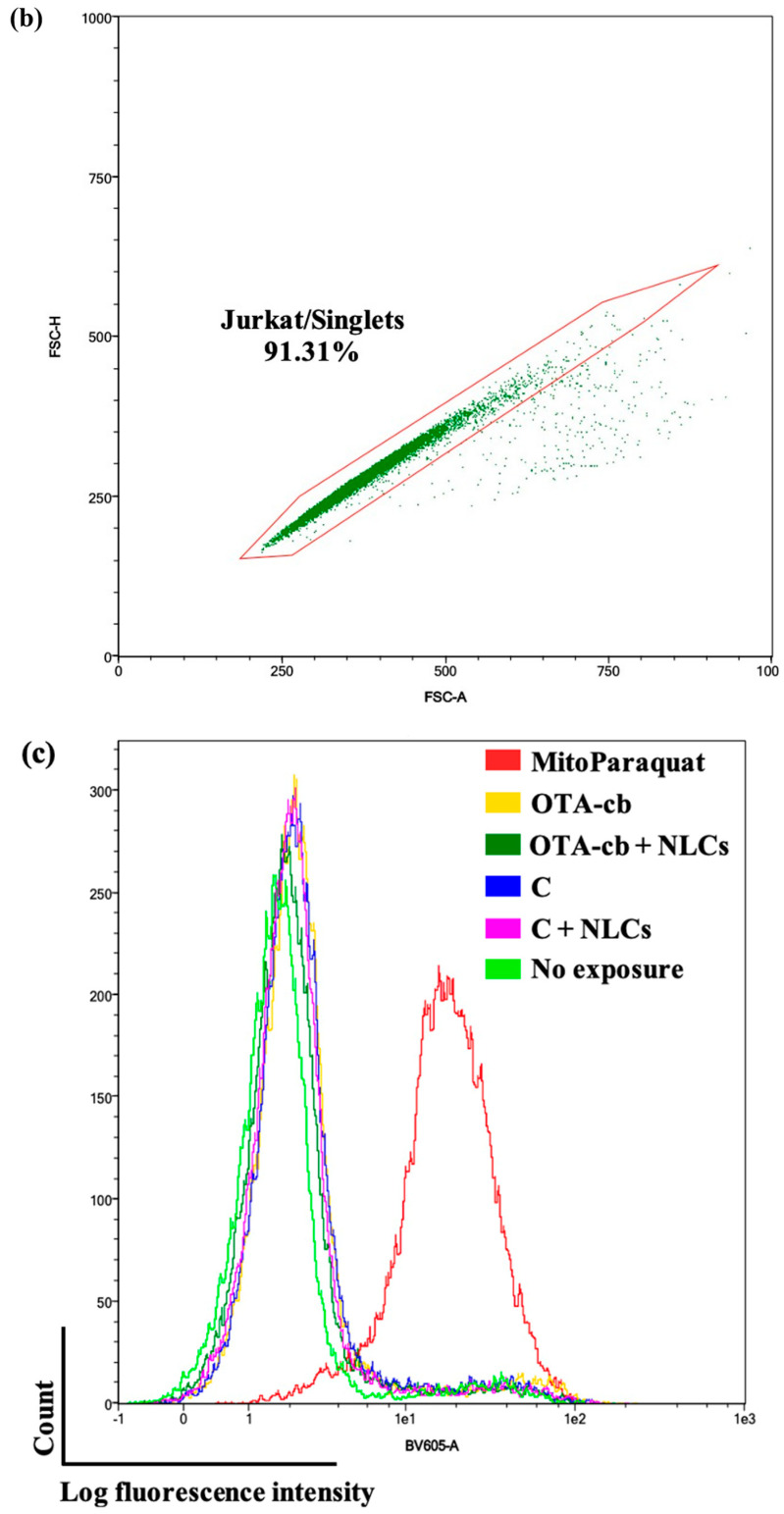

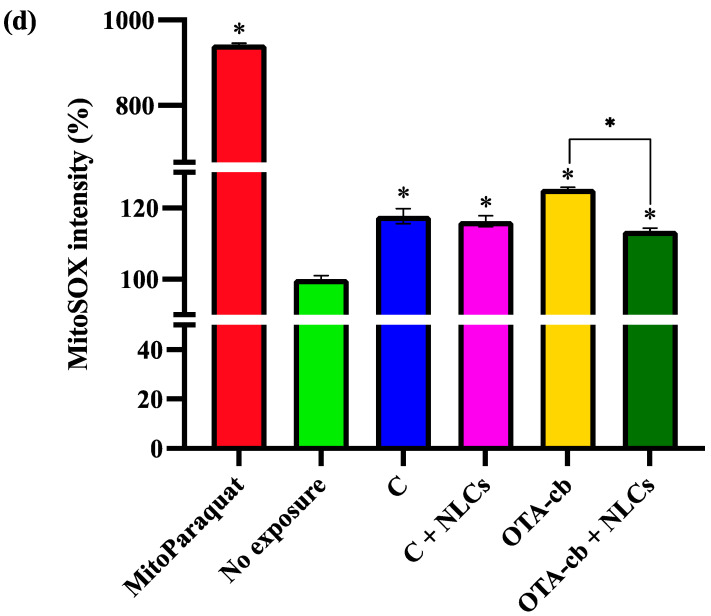
MitoSOX-based flow cytometry was employed to detect mitochondrial reactive oxygen species (ROS) in Jurkat cells following exposure to intestinal digests (0.330 ± 0.004 μM OTA). The process involved several steps to obtain the intensity of the MitoSOX fluorescence from the Jurkat singlets: (**a**) identification of viable Jurkat cells, based on side scatter (SSC) and forward scatter (FSC) characteristics, displayed in density plots; (**b**) gating of the singlet Jurkat cells based on forward scatter characteristics; (**c**) median intensity determination of the fluorescence of the Jurkat singlets acquired using the B4 channel after exposure; (**d**) bar graph reporting the mean MitoSOX intensity ± SD (n = 4). Significative differences are indicated for *p* < 0.05 (*). Significant differences between nonexposed cells and cell exposed to MitoParaquat, C, C + NLC, OTA-cb, and OTA-cb + NLC digest are denoted by asterisks directly above the columns. Significative differences for OTA-cb vs. OTA-cb + NLCs are indicated by asterisks above the brackets. C, control; NLCs, nanostructured lipid carriers; OTA, ochratoxin A; OTA-cb, ochratoxin-A-contaminated bread.

Remarkably, exposure to each tested intestinal digest resulted in a significant increase in mitochondrial ROS levels compared to unexposed cells (*p* < 0.0001) (Figure 4). The increases in mitochondrial ROS production were 17.7 ± 2.1% and 25.3 ± 0.5% for the C and OTA-cb intestinal digest exposures, respectively, compared to the nonexposed cells. However, the presence of NLCs, which did not affect the level of mitochondrial ROS in the C + NLC intestinal digest, resulted in a significant reduction (*p* < 0.001) of mitochondrial ROS levels in the OTA-cb + NLC intestinal digest (13.4 ± 0.6%) compared to the ROS levels obtained after the cells’ exposure to the OTA-cb digest, demonstrating an important antioxidant effect at the mitochondrial level.

### 3.10. Mitochondrial Mass Analysis

**Figure 5:**
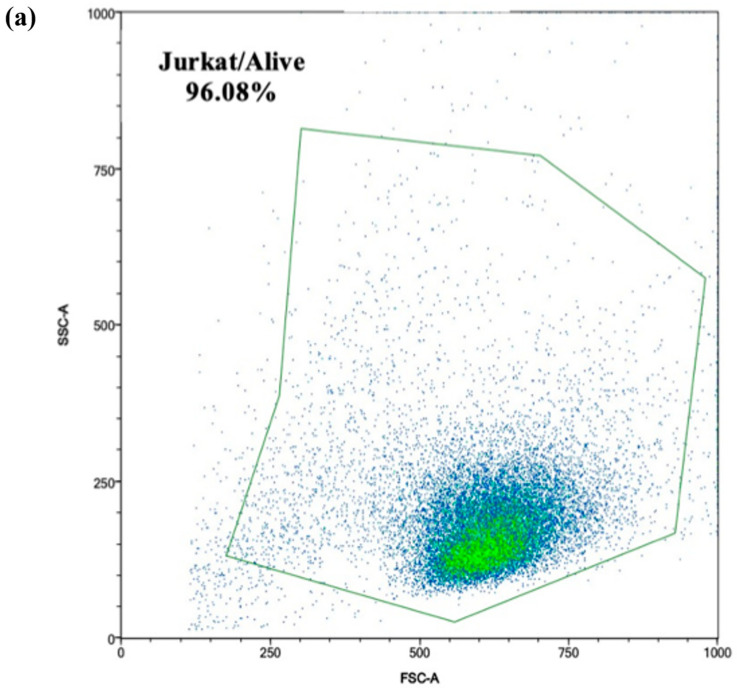

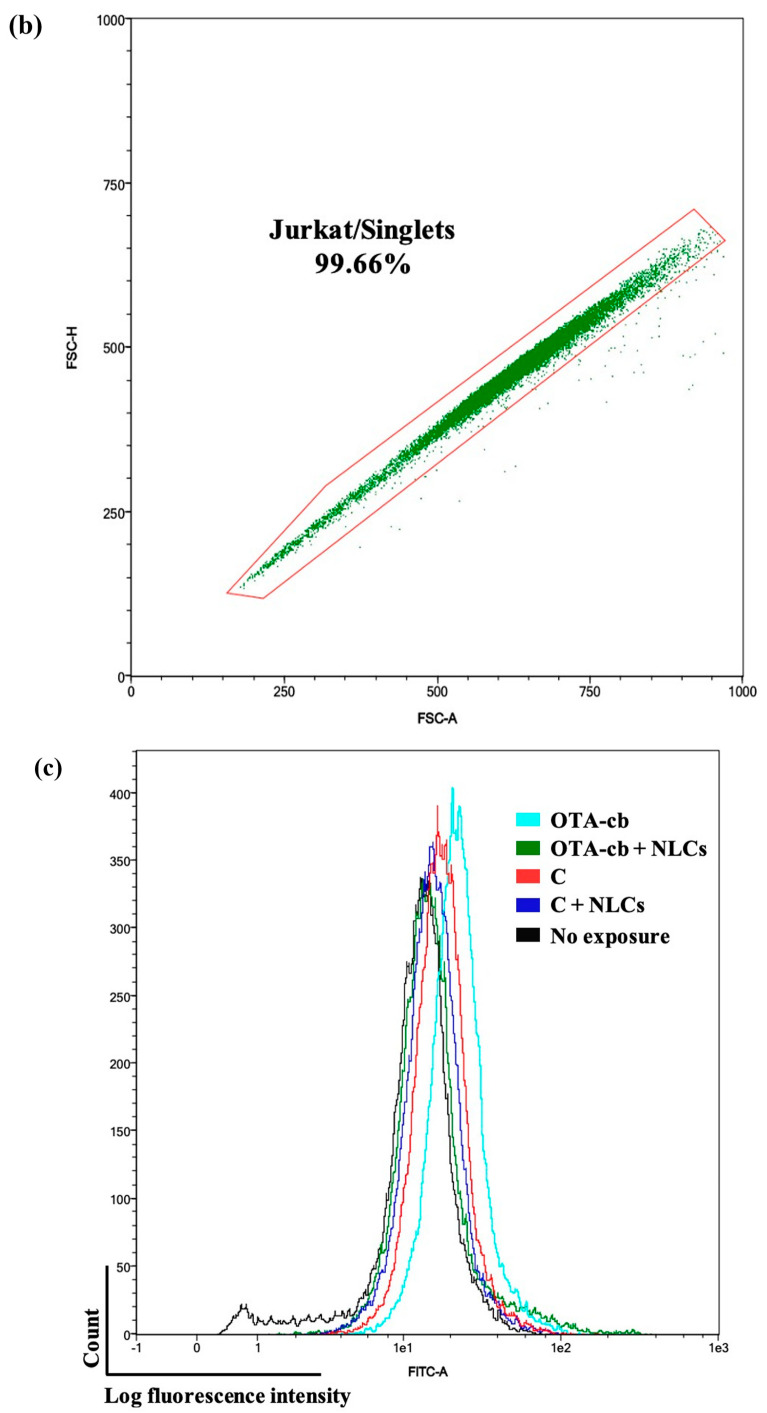

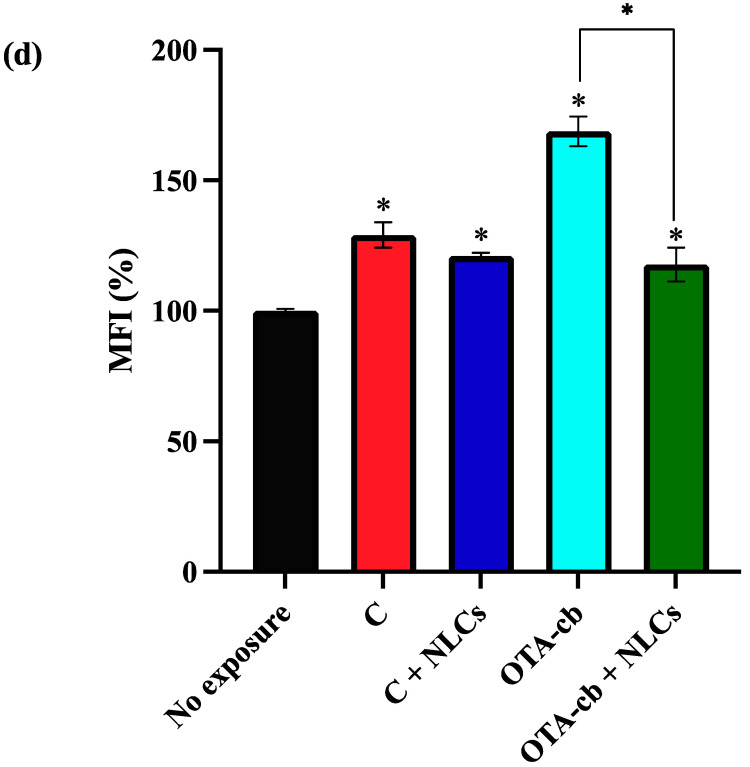
The impact of intestinal digests (0.330 ± 0.004 μM OTA) on the mitochondrial mass was evaluated. Several steps were undertaken to obtain the median fluorescence intensity (MFI) from the Jurkat singlets: (**a**) identification of the viable Jurkat cells based on side scatter (SSC) and forward scatter (FSC) characteristics; (**b**) gating of the singlet Jurkat cells using forward scatter characteristics, illustrated in density plots; (**c**) determination of the MFI of the Jurkat singlets obtained using the FITC channel; (**d**) bar graph reporting MFI ± SD (n = 4) of the MitoTracker dye following 7 days of incubation of the Jurkat cells with intestinal digests. Significative differences are indicated for *p* < 0.05 (*). Significant differences between the nonexposed cells and cells exposed to the C, C + NLCs, OTA-cb, and OTA-cb + NLCs digests are denoted by asterisks directly above the columns. Significative differences for OTA-cb vs. OTA-cb + NLCs are indicated by asterisks above the brackets. C, control; NLCs, nanostructured lipid carriers; OTA, ochratoxin A; OTA-cb, ochratoxin-A-contaminated bread.

For both the C and OTA-cb intestinal digests, significant increments in the mitochondrial mass of 27% (*p* < 0.01) and 69% (*p* < 0.001), respectively, were observed (Figure 5). Interestingly, when the NLC formulation was present in the intestinal digest, the observed increase in the mitochondrial mass for the OTA-cb + NLC digest was only 17%, which is very similar to the increase observed for the C + NLC intestinal digest (21%).

## 4. Discussion

The primary aim of this study was to determine whether carotenoids encapsulated in NLCs influence the bioaccessibility and bioavailability of OTA and mitigate its toxic effects on human gastrointestinal and lymphocyte cell lines, following an in vitro digestion simulation. The results in the present study did not show significant differences regarding OTA bioaccessibility between the gastric digests of OTA-cb alone and OTA-cb with NLCs (Table 1). This trend was also observed in the intestinal phase. This indicates that the NLC formulation did not reduce OTA bioaccessibility during in vitro digestion. Regarding the possible influence of carotenoids on the bioaccessibility of OTA, few studies have been reported. In particular, Escrivá et al. [18] evaluated the bioaccessibility of OTA in bread enriched with only lyophilized pumpkin pulp. The pumpkin pulp in the contaminated bread reduced OTA bioaccessibility in the intestinal phase to 70% starting from an initial bioaccessibility of 88%, measured after the digestion of contaminated bread without the functional ingredient. The situation described by Escrivá et al. [18] does not perfectly apply to the context of the following study. In their case, pumpkin pulp was a functional ingredient of the bread, whereas, in our case, the NLC formulation with the carotenoid extract was a supplement that was externally combined with the bread contaminated by OTA.

A significant difference was observed between the gastric and intestinal concentrations of OTA, and these results suggest that OTA’s release occurs mainly during the intestinal phase of digestion, likely due to the physicochemical properties of OTA and the pH shift from 3 to 7, which promote the release of OTA from the bread [18]. Considering the bioaccessibility values obtained, the results are in line with other works in which the OTA bioaccessibility was assessed using different food matrices, such as baby food [19] and buckwheat [20], with values above 85%.

Regarding bioavailability, which was assessed using a simulated human gastrointestinal barrier with Caco-2 cells, similar results were observed. The presence of the NLC formulation in the intestinal digest did not reduce or influence the absorption and diffusion of OTA from the apical to the basolateral compartment in the Caco-2 cells. These results are in line with those of Sergent et al. [21], who found that 15–20% of the initial OTA amount in the apical compartment reached the basolateral compartment unaltered after 3 h. Based on the results for the bioaccessibility and bioavailability of the OTA-cb and OTA + NLCs, it can be concluded that neither the NLC formulation nor the carotenoids interfered with OTA bioaccessibility. Further investigations are needed to determine whether varying amounts of the NLC formulation can influence OTA bioaccessibility and bioavailability.

Differentiated Caco-2 cells were also used to evaluate cell viability after exposure to the OTA-cb and OTA + NLC intestinal digests. The results show that individual OTA exposure affects the cell viability in a concentration dependent manner, with the highest reduction in cell viability observed for the non-diluted intestinal digest (3.63 ± 0.08 µM), corresponding to reductions of 18% after 24 h and 21% after 48 and 72 h. Considering the initial concentration of OTA in the intestinal digest, these findings are in line with the results reported by several studies. MTT assay data show a concentration-dependent cytotoxic action of OTA in differentiated and undifferentiated Caco-2 cell lines at different exposure times (24, 48, and 72 h) [22,23,24]. Romero et al. [23] showed a reduction in undifferentiated Caco-2 cells’ viability of nearly 20% when exposed for 24 h to OTA in the concentration range of 1–3 µM. Cano-sancho et al. [22] and Sobral et al. [24] also observed reductions in Caco-2 cell viability (~25%), with concentrations of OTA between 0.5 and 160 µM and 0.625 and 20 µM after 48 and 72 h of exposure, respectively. Concerning the possible cytoprotective effect of NLCs within the OTA-cb + NLC intestinal digest, significant increases in the cell viability of 21–35% at 48 h (*p* < 0.01) and of 9–12% at 72 h (*p* < 0.01) were observed. These results suggest how the presence of carotenoids in the intestinal digest could help reduce OTA’s cytotoxic effects and improve cell viability. Similar results, even if against beauvericin (BEA), were also observed by Juan-García et al. [25], who evaluated the cytoprotective effect of Goji berry extract (GBE) and two distinct carotenoids, lutein (LUT) and zeaxanthin (ZEA), using Caco-2 cells. Interestingly, those results showed how singularly GBE, LUT, and ZEA were not cytotoxic for Caco-2 cells and were able to improve cell viability, especially in the case of GBE, for which a cell viability of over 100% was recorded, especially for the 48 h exposure time frame. In addition, GBE, LUT, and ZEA showed an ability to improve cell viability when employed in the co-exposure with BEA and the pre-treatment experiment, counteracting mycotoxin cytotoxicity [25]. Also, Frangiamone et al. [26], who exposed SH-SY5Y cells to an intestinal digest of AFB1, as well as OTA-contaminated bread enriched with pumpkin extract, observed that the pumpkin extract itself was able to improve cell viability after a 72 h exposure time.

Concerning the cell-cycle analysis, the presence of OTA in the OTA-cb and OTA-cb + NLC intestinal digests promoted a shift in cell distribution through the sub-G0/G1 phases compared to the C and C + NLC intestinal digests. These results can be justified considering the tendency of OTA to induce cell death through cell-cycle disruption. No other important differences were observed when comparing the OTA-cb and OTA-cb + NLC intestinal digests with the control. Even though a previous study has demonstrated that exposure to high doses of OTA (3.1–40 µM) increases the arrest of the cell cycle in the G0/G1 phases when compared to the control [27,28], it is possible that the OTA concentration in this study was not sufficient to disrupt Jurkat cell cycles. For C + NLCs, an important reduction in the percentage of cells in the G2/M phases was observed suggesting how the presence of carotenoids is responsible for an arrest of the cell cycle, also justified by the increases in the number of cells in the G0/G1 and the sub-G0/G1 phases. Similar results were reported by Gloria et al. [29], after exposing human breast adenocarcinoma cell lines (MCF-7 and MDA-MB-235) to different concentrations of lycopene and β-carotene. In fact, a reduction in the percentage of cells in G2/M phases with a consecutive increase in the percentage of cells in the G0/G1 phases was reported.

As regard the results of the apoptosis/necrosis pathway’s evaluation, significant increases in the percentage of death cells were recorded for the OTA-cb (*p* < 0.001) and OTA-cb + NLCs (*p* < 0.01) treatments compared to the C digest. The increases in the percentage of death cells recorded for both treatments could be related to the presence of OTA in the intestinal digest. Different studies have demonstrated that OTA exposure in different cells lines results in an increase in the amount of apoptotic and necrotic cells. Zhao et al. [30] observed significant increases in the apoptotic rate and in the number of apoptotic Het-1A cells when exposed to different concentrations of OTA (5-10 µM) for 24 h. Similar results on porcine alveolar macrophages (PAMs) were also reported by Xu et al. [31] and Gan et al. [32]. In these studies, exposure to OTA for 48 h (0.1–2 µg/mL [31] and 5–7 µM [32]) led to increases in the late apoptotic cells and apoptotic rate. The low value of apoptotic cells reported in the present work could be explained considering the lower concentrations of OTA in the intestinal digest, which, similar to the cell-cycle analysis, was not sufficient to exert a significant effect on Jurkat cells.

In addition to investigating the effects of OTA on the cell-cycle and apoptosis/necrosis pathway, this study also focused on one of the main mechanisms through which OTA exerts its cytotoxic effects, as follows: the promotion of free-radical-mediated oxidative damage, which is strongly associated with the carcinogenicity and cytotoxicity of this particular mycotoxin [33]. Costa et al. [34] evidenced how even after a very short period of exposure (30 min) to high dose of OTA (50 µM), Vero-E6 cell line showed an important increase in the intracellular ROS level. Xu et al. [31] observed that the exposure of PAMs to OTA (0.1–2 µg/mL; 48 h) was correlated with an increased intracellular oxidative stress. In particular, an increased level of cytoplasmatic ROS, ascribable to the upregulation of the ROS-relative TLR4/MyD88 signaling pathway, was assessed. Nevertheless, for the sole increase in the intracellular ROS level that Liu et al. [35] were able to observe in human blood mononuclear cells, the OTA exposure (5–20 μM; 24 h) was correlated to lower antioxidant activity by glutathione. This tripeptide is strongly involved in the counteraction of oxidative stress promoted by free radicals. An exacerbated oxidative stress in the cellular compartment can also lead to an important alteration of mitochondrial functions.

Even though ROS production mainly occurs in mitochondria during oxidative phosphorylation in the electron transport chain (ETC); mitochondria themselves represent one of the main targets of ROS intracytoplasmic accumulation. Zhao et al. [30] observed an important alteration in the mitochondrial function following the exposure of human epithelial Het-1A cells to OTA (2.5–10 µM; 24 h). Specifically, a decline in the mitochondrial membrane potential (MMP) and a reduction in activity of mitochondrial respiratory chain complex 1 (MRCC1), ascribable to the increased ROS level after OTA exposure was observed. Moreover, increased lipid peroxidation of mitochondrial membrane, justified by the high levels of intracellular malondialdehyde (MDA), a biomarker of oxidative stress directly correlated with the degree of lipid peroxidation, was also observed. Similar results were also observed by Li et al. [36] in GES-1 cells after 24 h exposure to OTA (5–20 µM). A high level of cytoplasmatic ROS and decreased expression of SOD2 and HO-1 were linked to a reduction in MPP, causing permeability dysfunction on the mitochondrial membrane. Consequently, OTA can induce apoptosis promoting and stimulating the mitochondrial apoptosis pathway.

Interestingly, in addition to inducing mitochondrial function disturbance and causing apoptotic cell death, the results showed that OTA was responsible, at the same time, for inducing mitochondrial biogenesis, resulting in increased mitochondrial mass and volume [36]. Mitochondrial permeability transition pore (MPTP), which is directly correlated with MPP dysfunction, was also observed by Wang et al. [37]. Cytochrome C (cyt-c) release and caspase-3 activation in IPEC-J2 cells exposed to OTA (2–8 µM; 12 h) resulted in a higher percentage of apoptotic cells. Furthermore, Wang et al. [37] suggested that the major site of intracellular ROS production after OTA exposure could be the mitochondria itself. In fact, OTA exposure significantly increased the mitochondrial ROS level, which could explain the MPTP, the subsequent increase in the intracellular ROS level, and activation of the apoptotic pathway. Therefore, the mechanism of action of OTA for inducing apoptosis in IPEC-J2 cells is an ROS-dependent mitochondrial apoptosis pathway.

Aligned with the aforementioned finding, in the present work OTA exposure was responsible for a significant rise in intracellular (*p* < 0.001) and mitochondrial ROS (*p* < 0.001), as well as a significant increase in mitochondrial mass (*p* < 0.001). Figure 3 illustrates the intracellular ROS level observed after exposure of the Jurkat cells to the different intestinal digests. The results show that the exposure of Jurkat cells to the C and OTA-cb intestinal digests increased the intracellular ROS levels, especially in the case of the OTA-cb intestinal digest. Interestingly, in both C + NLCs and OTA-cb + NLCs, the presence of nano-encapsulated carotenoids reduced intracellular ROS levels, restoring them to unexposed cell levels. The ability of carotenoids within NLCs formulation to counteract cytotoxic effect of OTA was also observed for MitoSOX and MitoTracker analysis (Figure 4 and Figure 5). In both cases, exposure of Jurkat cells to the OTA-cb intestinal digest, led to a significant increase in mitochondrial ROS level (*p* < 0.001) and mitochondrial mass (*p* < 0.001), a result aligned with the aforementioned works [30,36,37]. Furthermore, in the OTA-cb + NLC intestinal digest, the presence of NLCs helped reduce mitochondrial ROS levels and mitigate mitochondrial mass increase. All these results collectively confirm the formulation’s ability, through its carotenoids, to counteract OTA toxicity in vitro.

The mechanism through which carotenoids might be able to limit the damage promoted by OTA can be attributed to their ability to physically and chemically quench singlet oxygen (^1^0_2_) and to effectively scavenge other ROS, such as superoxide anion, hydroxyl radical, and hydrogen peroxide [38]. The antioxidant action of carotenoids was observed at the cytoplasmatic level but also directly in the mitochondria. Usually, carotenoids, once they have entered the cells and bonded to the carotenoid-binding protein, are able to integrate into the mitochondrial membrane. From there, carotenoids can intervene against mitochondrial ROS or become substrates of β-carotene oxygenase 2 (BCO-2) to be converted into apo-carotenoids and, subsequently, into retinal by β-carotene oxygenase 1 (BCO-1) [39]. Furthermore, carotenoids and the retinal itself can be directly transported to the nucleus and influence gene expression by interacting with transcriptional factors and nuclear receptors. In this way, they regulate the expression of anti-inflammatory, antioxidant, and anti-apoptotic genes [39,40]. Frangiamone et al. [41] evaluated the potential effect of a pumpkin–fermented whey (P-FW) mixture against AFB1 and OTA immune toxicity in human lymphoblastic Jurkat cells using a transcriptomic approach. Especially for OTA, it was observed that the functional ingredients in the digested bread downregulated the gene expression of JUN/FOS, MAPK and NF-kB, thereby counteracting the overexpression of Myd88 promoted by OTA exposure, limiting the IFN pathway’s activation, and promoting an anti-inflammatory effect.

Overall, the results of the MTT assay on the Caco2 cells and the flow cytometry on the Jurkat cells confirm the ability of the carotenoids contained within the NLC formulation to counteract the cytotoxic effects of OTA, improving cell viability, limiting the production of intracellular and mitochondrial ROS, and counteracting critical alterations in mitochondrial mass. The ability of carotenoids to exert these effects, after undergoing an in vitro digestion process, can be correlated with their encapsulation within the NLCs. The encapsulation of compounds such as carotenoids into nanocarriers is reported to improve their bioaccessibility and stability, especially within the gastrointestinal tract during the digestive process [12].

## 5. Conclusions

In this work, the effects of carotenoid-loaded NLCs on OTA’s bioaccessibility, bioavailability, and cytotoxic effects were investigated using a simulated in vitro digestion protocol. The results show that OTA is involved in the promotion of cell death, cytoplasmatic and mitochondrial ROS generation, and mitochondrial dysfunction. Even though the presence of the NLC formulation was not able to reduce the OTA bioaccessibility and bioavailability, it was possible to demonstrate a protective role against cytotoxicity induced by OTA in human cells, especially by mitigating oxidative stress both at cytoplasmatic and mitochondrial levels. Also, the ability to counteract mitochondrial mass changes was observed, suggesting how carotenoids are capable of intervening directly on the mitochondrial compartment.

Overall, these findings suggest that the formulation itself preserved the chemical structures of the encapsulated carotenoids and their antioxidant activity, making them able to directly interfere with OTA-induced cytotoxicity. Further research is needed to better understand the mechanism of action of carotenoids and to explore potential industrial applications for this type of nanoformulation using in vivo studies.

## Figures and Tables

**Table 1:** Recipes for the control bread (C) and ochratoxin A-contaminated bread (OTA-cb).

Ingredient	C (g)	OTA-cb (g)
Wheat flour	63.5	56.5
Contaminated barley flour (OTA)	0	7
Water	33	33
Salt	1.3	1.3
Sugar	2	2
Fresh yeast	4	4
Total quantity	103.8	103.8

**Table 2:** OTA concentrations of in vitro gastric and intestinal digests of contaminated bread without and with NLCs (mean ± SD, n = 3).

Sample	Gastric Concentration (µM)	Intestinal Concentration (µM)
OTA-cb	0.13 ± 0.00	3.63 ± 0.08
OTA-cb + NLCs	0.12 ± 0.00	3.30 ± 0.04

NLCs, nanostructured lipid carriers; OTA-cb, ochratoxin-A-contaminated bread.

**Table 3:** Cell viability after OTA-cb and OTA-cb + NLC exposure for various times and dilutions. The cell viability (%) results obtained for differentiated Caco-2 cells after exposure to OTA-cb and OTA-cb + NLC intestinal digests. Data are expressed as the means ± SD (n = 4). Significant differences were determined between cells exposed to the OTA-cb and OTA-cb + NLC intestinal digests at equivalent dilutions and exposure durations. The means denoted by asterisks indicate significant differences between treatments performed at equivalent dilutions and exposure durations. (*p* < 0.05).

Cell Viability (%)						
Exposure Time	24 h		48 h		72 h	
Intestinal Digest	OTA-cb	OTA-cb + NLCs	OTA-cb	OTA-cb + NLCs	OTA-cb	OTA-cb + NLCs
*1/32*	97 ± 3	101 ± 3	98 ± 3 *	119 ± 4 *	98 ± 1 *	110 ± 4 *
*1/16*	97 ± 5	103 ± 4	95 ± 1 *	122 ± 2 *	103 ± 1 *	113 ± 3 *
*1/8*	100 ± 4	104 ± 3	97 ± 3 *	123 ± 1 *	105 ± 3 *	114 ± 3 *
*1/4*	96 ± 4	103 ± 6	104 ± 1 *	126 ± 4 *	107 ± 3 *	119 ± 6 *
*1/2*	97 ± 2	101 ± 5	101 ± 5 *	123 ± 3 *	105 ± 3 *	116 ± 3 *
*No dilution*	82 ± 2	89 ± 4 *	79 ± 5 *	115 ± 4 *	79 ± 3 *	89 ± 1 *

NLCs, nanostructured lipid carriers; OTA-cb, ochratoxin-A-contaminated bread.

## Data Availability

The data are contained in the article.

## Supplementary Materials

The following supporting information can be downloaded at: https://www.mdpi.com/article/10.3390/foods13213351/s1, Figure S1. SEM photomicrographs of NLCs.
