# Supplementary material for: Protective Effects of Carotenoid-Loaded Nanostructured Lipid Carriers Against Ochratoxin-A-Induced Cytotoxicity
Source: Foods. 2024 Oct 22;13(21):3351. doi: 10.3390/foods13213351 (PMC11545611; doi:10.3390/foods13213351)
Supplement: Supplementary file 1 [file foods-13-03351-s001.zip › foods-3227351-supplementary.pdf]

Supplementary material

NLCs morphology determination

Before lyophilization, NLCs morphology was evaluated through Scanning Electron Microscopy (SEM). A microscope slide disk was firstly fixed on an aluminium specimen stub covered with a double-sided adhesive carbon disc, then fresh nanoparticle suspension was diluted and deposited on the microscope slide. Chromium was sprayed on the samples before imaging (100 mA, 22 s, 8 nm thickness) (Quorum Q150T ES East Grinstead, West Sussex, UK).

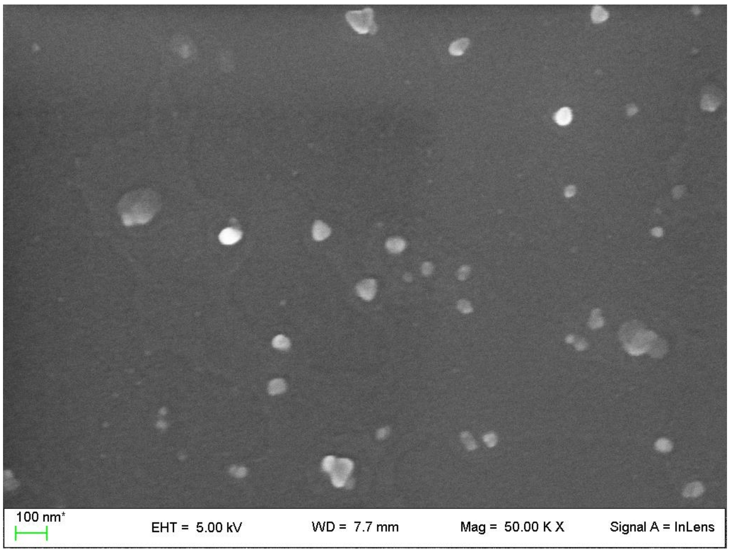

Figure S1. SEM photomicrographs of NLCs
